# Dental Department of University of Maryland

**Published:** 1882-04

**Authors:** 


A Dental Department of the University of Maryland (we learn before going to press) has just been perfected, and will begin its regular session next fall. This is another recognition of dentistry as a specialty of medicine. As this is one of our "hobbies," our friends need not be surprised that we greet this new department added to this time-honored school of medicine with a hearty welcome. We warmly congratulate the faculty of the university on being able to secure Prof. F. J. S. Gorgas, M.D., D.D.S., in the chair of Principles of Dental Science, Dental Surgery and Mechanism, and Prof. Jas. H. Harris, M.D., D.D.S., in the chair of Operative and Clinical Dentistry. Prof. Gorgas, so long and favorably known as the Dean of the "Old" Baltimore College of Dental Surgery is an amiable gentleman and a most excellent teacher. He presided in his official duties during our two courses of lectures in this "Old" school and from his hand we received our diploma with much pride. We afterward became an instructor in the same institution with him for a term of six years, and found him zealous in all his work. Prof. Jas. H. Harris has been a clinical instructor and Professor of Dentistry in the same "Old" dental college since 1871. We have been side by side over our chairs (private and in the clinic), for years, and we know him well to love him better. He never tires to do a favor or instruct a student. He has no superior as a dental operator, and is not wedded to any one gold, or mode of operating.

Since writing the above we have received the circular announcement, and see that John C. Uhler, M.D., D.D.S., is made Demonstrator of Mechanical Dentistry; he is also another extract from the faculty of the "Old" Baltimore Dental School, where he has competently officiated for the past six or eight years. He is a cheerful instructor, and ever on the alert for something new. Frank L. Harris, D.D.S., Demonstrator of Operative Dentistry, is a good operator, and a younger brother of Prof. Jas. H. There will be four assistant Demonstrators. The other chairs of the Dental Department are filled by the able members of the Medical Faculty.

### THE CURIOSITIES OF HEREDITY.

We trust that none of our readers will forego the pleasure of perusing the very interesting article on "Birth and Breeding," which we print in our *Popular Science Department.* The results obtained by Mr. Holland are so curious, and so much at variance with the popular notions current on the subject, that the facts presented—obtained as they have been by the numerical method—will command the attention of all thoughtful readers.

The delay of the April Issue is caused by moving our printing-office. Several papers and pamphlets for notice, we are sorry to say, were lost in the move. Numerous letters are also unanswered, but shall now have prompt attention.

